# Physiological and molecular insights into nitrogen rate and planting density interactive regulation of black sesame nitrogen use efficiency, growth, yield, and seed quality

**DOI:** 10.1186/s12870-026-08114-8

**Published:** 2026-01-22

**Authors:** Min Wang, Xuefei Tian, Guangwei Wei, Huiyi Yang, Xiaohui Wang, Xi Yang, Sheng Fang, Ziming Wu

**Affiliations:** Key Laboratory of Crop Physiology, Ecology, and Genetic Breeding, Ministry of Education/College of Agronomy, Jiangxi Agricultural University, Nanchang , 330045 China

**Keywords:** Sesame, Planting density, Nitrogen metabolism, Gene expression, Seed quality

## Abstract

**Background:**

Optimizing nitrogen (N) application and planting density is a green and efficient agronomic strategy to increase crop yield and nitrogen use efficiency (NUE). However, the sesame responses to the interactive effects of N dose and planting density have not been fully elucidated. Here, we investigated the effects of different planting densities and N rates on the growth performances, physiological traits, N and carbon metabolism, yield components, and seed quality of two black sesame varieties (JHM and PYH). Two-year field experiments were conducted combining three planting densities (110,000, 160,000, and 330,000 plants.ha^− 1^) and three nitrogen rates (45, 90, and 135 kg.ha^− 1^).

**Results:**

Analyses revealed that sesame’s response to the combined effects of planting density and N rate is developmentally regulated and varietal-specific. Notably, we found that a moderate nitrogen dose of ≤ 90 kg.ha⁻¹ coupled with dense planting of ⁓330,000 plants per hectare improved sesame growth, NUE, yield, and seed quality. This optimal planting condition significantly improved N allocation to seeds, maximized the yield of JHM and PYH to 3.47 t·ha^⁻1^ and 3.84 t·ha^⁻1^, respectively, and considerably enhanced seed oil and unsaturated fatty acid contents. *AMT1* and *NRT2.13 A* were identified as promising candidate regulatory genes of sesame NUE and modulators of N metabolism under dense planting density and moderate N rate. Other candidate N regulatory and sucrose metabolism-related genes were also identified.

**Conclusions:**

This study exposes the complexity of mechanisms underlying sesame response to dual effects of planting density and N rate, and identifies a potential optimal planting condition to improving yield per unit area and seed quality. This agronomic optimization is likely to reduce environmental risk compared with high-N regimes. However, the proposed regime is optimal for the two tested varieties and the specific soil–climate conditions studied, and it should be validated in additional germplasm and environments before broader extrapolation. .

**Supplementary Information:**

The online version contains supplementary material available at 10.1186/s12870-026-08114-8.

## Background

Black sesame seeds have long been among the most valued oilseeds in East-Asian traditional medicine for their marked nutritional value and pharmacological properties [1, 2]. They are considered elite traditional health foods and contain numerous nutrients and health-promoting compounds, including proteins, lignans, flavonoids, lipids, phenolic acids, and minerals, making them essential resources for diversified applications in the food industry [2]. The current human demand for natural, functional foods has expanded the market for black sesame products. As a consequence, black sesame has become a crucial cash crop for smallholder farmers, particularly in hilly regions of southern China [3, 4]. However, in the past 10 years, sesame cultivation in China has faced many challenges, such as reduced availability of arable lands and ongoing agricultural restructuring, resulting in steady production [5, 6]. Nitrogen fertilization rates up to 150 kg.ha^⁻1^ have been adopted to improve yield performance in black sesame cultivation systems. However, nitrogen use efficiency (NUE) in these systems remains suboptimal, ranging from 17.8% to 32.5%, which is significantly lower than the average of 50%–70% observed in developed countries such as those in Europe and North America [7, 8]. In addition, some farmers in China transplant sesame at extremely wide spacing, believing that coupling low planting density with high N nutrition can improve the number of capsules per plant and seed yield. Yet, limited information is available to support this claim. Although this system may favor adequate N nutrition for the single plant [9], group effects are equally important for achieving high yields. Therefore, it is crucial to investigate the physiological, biochemical, and molecular changes in developing sesame plants under various nitrogen rates and planting densities.

Increasing planting density is an effective way to enhance yield and improve NUE. This concept has been studied and confirmed across a wide range of crops, including rice [10, 11], maize [12], wheat [13], and oilseed rape [14], among others. Recent studies have revealed that optimized plant densities can reduce N input while maintaining sesame yield potential. Specifically, increasing sesame planting density to 225,000–375,000 plants·ha⁻^1^ in red soil systems enables a reduction in N application rates to 105 kg·ha⁻^1^ without compromising grain productivity. However, under high-density planting regimes, the elevated sesame plant population reduces per-plant N availability, resulting in decreased leaf chlorophyll content and net photosynthetic rates. This N limitation concomitantly diminishes stem diameter and capsule number per plant, while promoting vertical growth and earlier initiation of capsules. Notably, these morphological trade-offs are offset by significant enhancements in leaf area index (LAI), population-level dry matter accumulation, and capsule number per unit land area. At the physiological maturity stage, N remobilized to kernels accounted for 33.0%–44.3% of total plant nitrogen uptake, highlighting the key role of N metabolism as a dominant sink during reproductive development. These findings indicate that planting density and N input are key factors influencing sesame growth and yield. However, their interactive regulatory mechanisms in sesame physiology and molecular processes remain poorly characterized.

Nitrogen uptake and distribution are closely linked to NUE in plants. Nitrate (NO_3_^−^) and ammonium (NH_4_^+^) are the two primary sources of nitrogen (N) in the environment for plants. Nitrogen uptake and translocation in plants are mediated and tightly regulated by tissue-specific transporters, including nitrate transporters (NRT1 and NRT2) [15], ammonium transporters (AMT) [16], and amino acid permeases (AAP) [17], as well as by coordinated expression of N assimilation-related genes, such as glutamine synthetase (*GS*) [18, 19]. In maize, under controlled N dose and planting density, higher expression of *AMT1;1b* in leaves and *AAP5* in both leaves and stem nodes during the grain-filling stage suppresses N remobilization to ears [20]. In contrast, induction of *AMT1;1a* and *AMT4* in stem nodes promotes N allocation to ears [20]. Preliminary investigations on seedlings have revealed that nitrogen-use-efficient sesame genotypes exhibit upregulated expression of NPF (Nitrate Transporter 1/Peptide Transporter Family) genes in root systems, concomitant with enhanced nitrogen accumulation across multiple tissues. Nitrogen is the most critical mineral nutrient required by plants. Its metabolism is tightly integrated with carbon metabolism, allowing proper growth and development of plants [21]. Photosynthesis depends on N channeled into enzymes, chlorophylls, and structural components of the photosynthetic machinery, while N uptake, transport and assimilation rely on reducing equivalents, ATP, and C-skeletons provided by photosynthesis [21, 22]. Hence, it is of utmost importance to dissect and understand the interactive regulation of N and C metabolisms in sesame by planting density and N doses.

In the present study, two widely adopted black sesame varieties in Jiangxi Province, China, were cultivated over two years under varying planting densities and N dose rates, and their growth performance, physiological traits, N and C metabolisms, yield components, and seed quality were assessed. Our objectives were to provide a physiological and molecular understanding of the interactive regulatory effects of plant density and N dose rate on sesame NUE, growth, production, and quality, and to identify appropriate planting densities and N doses for sustainable and improved sesame production. We hypothesized that (i) moderate N and high density will increase sesame NUE and yield; (ii) JHM and PYH may respond differently to varying planting densities and N dose rates; and (iii) specific N transporters and sucrose metabolism genes are associated with high NUE.

## Results and discussion

### Optimizing planting density and N rate stimulated sesame growth and photosynthesis

To explore the interactive effects of planting density and N application rate on sesame agronomic performance and quality, two varieties, ‘Jinhuangma’ (JHM) and ‘Poyanghei’ (PYH), were cultivated under a combination of different planting densities and N rates, including N45D11, N45D16, N45D33, N90D11, N90D16, N90D33, N135D11, N135D16, and N135D33. Where D11, D16, and D33 indicate planting densities of 110,000, 160,000, and 330,000 plants per hectare, respectively, and N45, N90, and N135 indicate nitrogen application rates of 45 kg, 90 kg, and 135 kg of N per hectare, respectively, in 2020 and 2023.

**Fig. 1:**
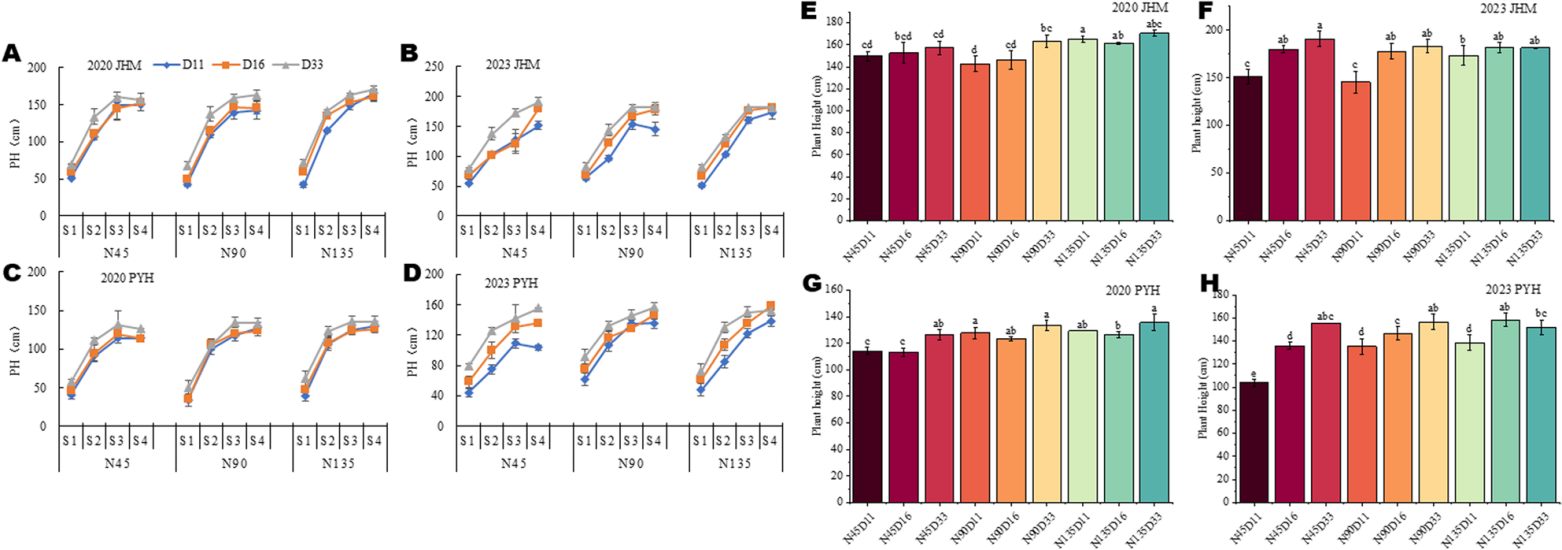
Interactive effects of nitrogen dose and planting density on plant height (PH) of black sesame in the years 2020 and 2023. **A**-**B** Dynamic PH changes of the variety JHM in 2020 and 2023, respectively. **C**-**D** Dynamic PH changes of the variety PYH in 2020 and 2023, respectively. **E**-**H** Variation of PH of JHM and PYH under different planting densities and N doses in 2020 and 2023. Note: N45, N90, and N135 represent the nitrogen application rate of 45, 90, and 135 kg · ha^− 1^, respectively. D11, D16, and D33, and D33 represent planting density of 110,000, 160,000, and 330,000 plants per ha^− 1^, respectively. S1 represents the initial flowering period, three days after topdressing; S2 represents 20 days after topdressing; S3 represents 40 days after topdressing; and S4 represents 60 days after topdressing

**Fig. 2:**
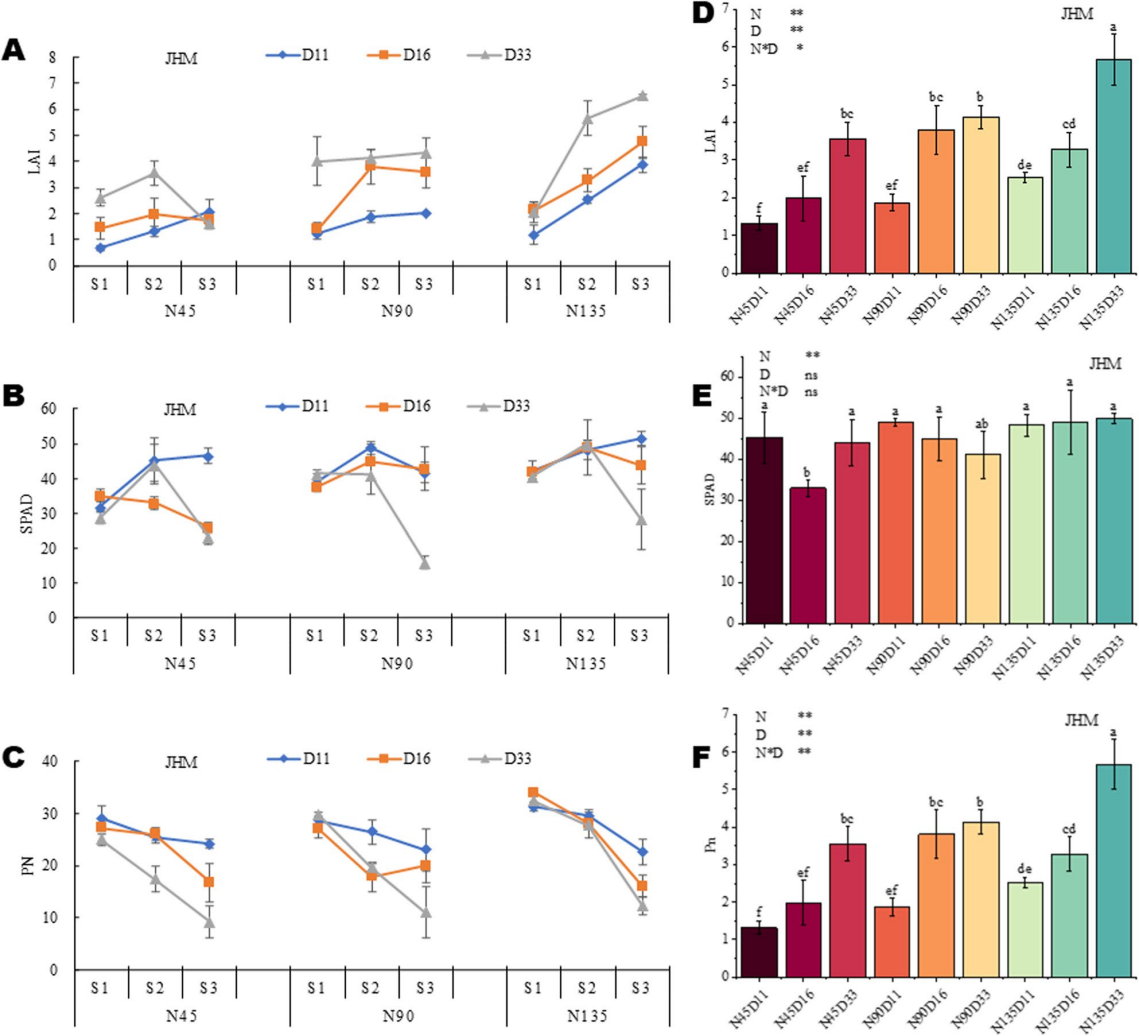
Interactive effects of nitrogen dose and planting density on leaf area index (LAI), chlorophyll content, and net photosynthesis (Pn) of black sesame variety JHM in 2023. **A** Dynamic LAI changes. **B** Dynamic chlorophyll content changes. **C** Dynamic Pn changes. **D**-**F** Variation of LAI, chlorophyll content, and Pn, respectively, under different planting densities and N doses. Note: N45, N90, and N135 represent the nitrogen application rate of 45, 90, and 135 kg · ha^− 1^, respectively. D11, D16, and D33, and D33 represent planting density of 110,000, 160,000, and 330,000 plants per ha^− 1^, respectively. S1 represents the initial flowering period, three days after topdressing; S2 represents 20 days after topdressing; S3 represents 40 days after topdressing; and S4 represents 60 days after topdressing

Plant height increased with development, reaching a maximum at S3 (late-flowering), under both combinations of planting densities and N rates (Fig. 1A-D). The interactive effects of planting density and N rate on plant height vary by sesame variety and year (growing environment) (Fig. 1E-H). The highest height for JMH was achieved under N135D11 in 2020 and under N45D33 in 2023 (Fig. 1E, F). Meanwhile, the highest height for PYH was recorded under N90D33 in both 2020 and 2023 (Fig. 1G, H). Similar to plant height, LAI (leaf area index) response to planting density and N rate varies depending on the variety (Fig. 2A, D and S2A, D). At the initial-flowering stage, LAI increased with increasing density. In contrast, at the mid and late-flowering stages, LAI was significantly influenced by planting density and N rate, particularly under N45 and N90 for JHM (Fig. 2A, D**)** and PYH (Figure S2A, D). The highest LAI was achieved under N135D33 for both varieties (Fig. 2D and S2D), indicating that increasing planting density and N rate increases LAI in sesame. These results show that sesame growth response to the simultaneous effects of planting density and N rate is developmentally regulated and genetically controlled. the responses may vary depending on the genetic background of the variety. Consistent with this, previous studies found that maize responses to the interactive effects of planting density and N rate are developmentally regulated and varietal-specific [23, 24].

Chlorophyll plays a critical role in light absorption and energy transformation in plants. The SPAD value reflects leaf chlorophyll content and nitrogen nutrient status in plant leaves [25, 26]. The SPAD value increased with higher in N rate and decreased with higher density. PYH and JHM both showed the highest SPAD values at the mid-flowering stage (Fig. 2B and S2B). No major differences in chlorophyll content were observed across planting densities and N rates (Fig. 2E and S2E). Net photosynthetic (Pn) rate reflects the ability of crops to assimilate carbon [27]. In accordance with SPAD values, Pn gradually decreased with maturity (Fig. 2C and S2C). Pn significantly decreased with increasing density. Dense planting increased competition for nutrients and light among individual plants, which led to a significant increase in plant height at early growth stages. The interactive effect of planting density and N rate on Pn varied depending on the variety (Fig. 2F and S2F). For instance, the highest Pn was recorded under N135D33, followed by N90D33 for the variety JHM (Fig. 2F). In contrast, for PYH, the highest Pn was observed under N135D16, followed by N135D11 (Figure S2F). A significant positive correlation was observed between chlorophyll content and Pn for the two varieties, while LAI and Pn showed a negative correlation (Figure S5). These results highlight the influence of planting density and N rate on interlinks between LAI, chlorophyll content, and photosynthesis, thereby promoting plant growth and crop productivity. As support, Zhang et al., found that increasing planting density significantly elevates LAI and the amount of intercepted photosynthetically active radiation, whereas it reduces Pn, stomatal conductance, and leaf chlorophyll content in maize [28]. Other studies have disclosed that increasing the N rate improve the vegetative status of plants, increase the effective radiation of the canopy, enhance the carbon assimilation ability of crops, and provide a material basis for increasing yield [29, 30]. In addition, Zhou et al., found that planting density positively regulate Pn at the early post-anthesis period, while the influence of N rate is mainly reflected at the late post-anthesis period [31]. Collectively, these findings underscore the need to optimize planting density and N application rate for improved plant production.

### Yield and yield components improved under moderate N rate with higher planting density

Sesame yield is primarily governed by three key yield components: capsule number per plant, seed number per capsule, and thousand-seed weight. Capsule number explained 62–74% of the yield variation across treatments, establishing it as the primary yield determinant [32, 33]. The number of capsules decreased with increasing density, and increased with higher N rate (Table S4). For both varieties, the highest capsule number per plant was achieved under N135D11 (Table S4). PYH maintained a higher capsule number under high-density conditions, indicating it is more suitable for dense planting. Table S5 presents a summary of the capsule logarithm and the capsule axis length. For JHM, nitrogen fertilizer had a significant effect on the capsule logarithm, which increased with N rate. As the density increased, the capsule logarithm decreased. When treated with N135D11 and N135D16, the capsule logarithm was the largest. The capsule axis length and the unit capsule node length increased with increasing density (Table S5). As for PYH, the capsule logarithm increased with N rate. Under the conditions of medium and low density, the capsule axis length increased with the increase in N rate. These results show that dense planting without appropriate N input will considerably reduce yield through dual effects of nutrient limitation and competition for light.

Sesame yield increased significantly with increasing planting density (Table S4). For JHM, the maximum yield of 2.95 t·ha^− 1^ and 3.47 t·ha^− 1^ was recorded in 2020 and 2023, respectively, under N90D33. Similarly, maximum yields for PYH (3.4 t·ha^− 1^ and 3.84 t·ha^− 1^, respectively, in 2020 and 2023) were recorded under N90D33. The lowest yield of 1.35 t·ha^− 1^ and 1.28 t·hm^− 2^ for JHM was recorded under N45D11 and N90D11, respectively. Previous studies have identified capsule number as the major trait that determines yield in sesame [32, 33]. However, we recorded the highest yield under N90D33, while the highest capsule number per plant was achieved under N135D11. This result suggests that the higher density has compensated for the population level capsule number. For example, in 2020, JHM exhibited 47.67 and 74.78 capsule numbers per plant under N90D33 and N135D11, respectively, suggesting that the population level capsule number under N90D33 (47.67 × 33000 = 822,580) might be significantly higher than under N135D11 (74.78 × 11000 = 1,573,110). PYH showed a 44.99% higher capsule number per plant under N90D33 than JHM, contributing to its 13.23% higher yield in 2020. Meanwhile, in 2023, PYH showed a 20.58% higher capsule number per plant under N90D33 than JHM, contributing to its 9.63% higher yield. These results further confirm the varietal-specific response to the combined effects of planting density and N rate in sesame. Moreover, they show that the genetic background of sesame varieties may underlie the plant’s physiological and molecular plasticity (e.g., root architecture, nutrient uptake efficiency) in response to density and N availability, thereby contributing to these differences.

Increasing planting density resulted in a significant decrease in single plant grain weight, but an increase in population yield (Table S4). Notably, yield responsiveness to elevated nitrogen inputs plateaued above 90 kg ha⁻¹, indicating marginal returns under high-N regimes. The thousand-grain weight increased with the increase in N rate, and decreased significantly with density. The total dry matter increases with increasing density and nitrogen application rate (Table S4). The harvest index was affected by nitrogen, and showed a decreasing trend with increasing nitrogen application rate. JHM exhibited maximum harvest index (HI = 0.26) under N90D11 treatment, representing 19% enhancement over the minimum HI values under N135D11 (HI = 0.21) in 2023. The maximum HI of PYH was 0.39 under N90D11, and the minimum was 0.27 under N135D16 in 2023 (Table S4). The HI is reduced under high N rate and planting density, suggesting limited nitrogen metabolism in sesame plants under higher N availability condition, thereby affecting carbon nutrition and overall productivity. Indeed, Nitrogen is the most important mineral nutrient for plant growth and is vital for the formation of seeds [34, 35]. Carbon and N are essential for plant growth and development, serving as the structural and functional backbone of organic compounds and driving crucial biological processes such as photosynthesis, carbohydrate metabolism, and N assimilation [36]. Under nitrogen-limited conditions (*N* ≤ 90 kg ha⁻¹), optimized planting density enhanced yield and HI, aligning with findings in maize [11]. Collectively, these findings indicate that ideal sesame yields can only be achieved under moderate N rate and adjusted higher planting density. Compared with 90 kg·ha^− 1^, 135 kg·ha^− 1^ did not significantly increase the biomass and the grain weight, suggesting low NUE.

### Significant translocation of N from other tissues to seeds in JHM than PYH

**Fig. 3:**
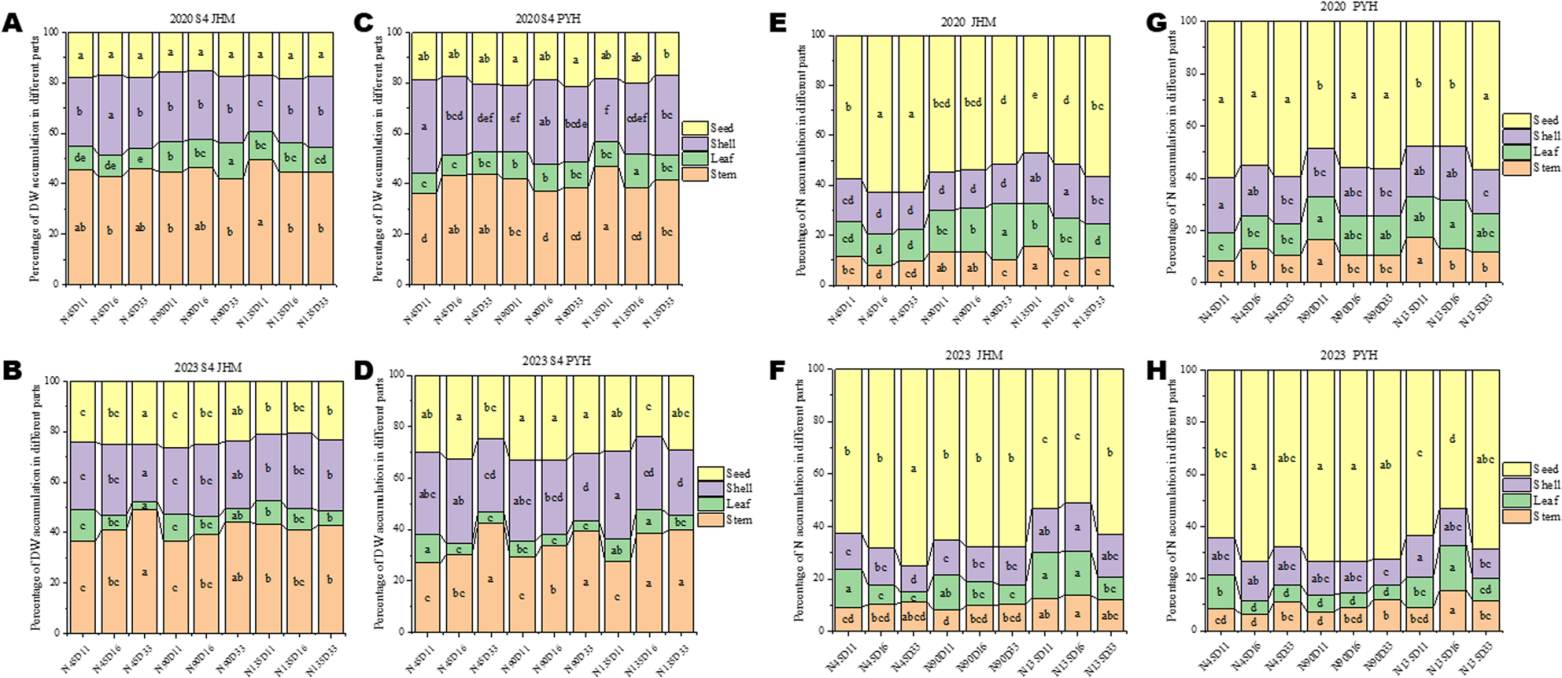
Interactive effects of nitrogen dose and planting density on dry matter distribution and N content of different sesame tissues at the maturity stage. **A**-**B** Dry matter distribution in tissues of variety JHM in 2020 and 2023, respectively. **C**-**D** Dry matter distribution in tissues of variety PYH in 2020 and 2023, respectively. **E**-**F** Nitrogen accumulation in different tissues of variety JHM in 2020 and 2023, respectively. **G**-**H** Nitrogen accumulation in different tissues of variety JHM in 2020 and 2023, respectively. Note: N45, N90, and N135 represent the nitrogen application rate of 45, 90, and 135 kg · ha^− 1^, respectively. D11, D16, and D33, and D33 represent planting density of 110,000, 160,000, and 330,000 plants per ha^− 1^, respectively. S1 represents the initial flowering period, three days after topdressing; S2 represents 20 days after topdressing; S3 represents 40 days after topdressing; and S4 represents 60 days after topdressing

Biomass partitioning at maturity followed the order stems > capsule shells > seeds > leaves (Fig. 3A-D). The highest seed biomass in PYH was observed under N90, while in JHM, it was observed under N45D33 and N90D33, supporting the conclusion that N90D33 might be the optimal planting density and N rate for higher seed yield in sesame. Nitrogen accumulation at maturity followed the order seeds > capsule shells > stems > leaves (Fig. 3E-H). The proportion of seed nitrogen in JHM decreased with increasing N rate, while the proportions in stem, leaves and capsule shells increased (Fig. 3E, F). With the increase in density, the proportion of seed nitrogen increased, while the proportion of leaf nitrogen decreased. Similar to JHM, the proportion of seed nitrogen in PYH decreased with increasing nitrogen application rate. But, in contrast the stems and capsule shells nitrogen increased slightly (Fig. 3G, H). N accumulation in seeds was highest in PYH under N90D33, N90D16, and N45D16 (Fig. 3G, H). In contrast, it was highest in JHM under N45D33 (Fig. 3E, F). These results confirm the conclusion of differential varietal response to the interactive effects of planting density and N rate in sesame. Differential response of cultivars to interactive effects of planting density and N rate has been observed in maize [24]. Dry matter allocation and N accumulation showed similar trends, confirming that moderate N input conditions of ≤ 90 kg ha⁻¹ may promote seed yield in sesame. It has been suggested that the senescence of vegetative tissues, especially the lower leaves, accelerates the transport of nitrogen from other tissues to grains [37, 38]. Therefore, we investigated changes in nitrogen accumulation in various tissues between the late-flowering and the harvesting. As illustrated in Figure S3, the change rates of nitrogen accumulation in seeds showed positive values, while in almost all other tissues, the change rates were negative. The results suggest a significant translocation of N from other tissues to seeds during maturity.

**Fig. 4:**
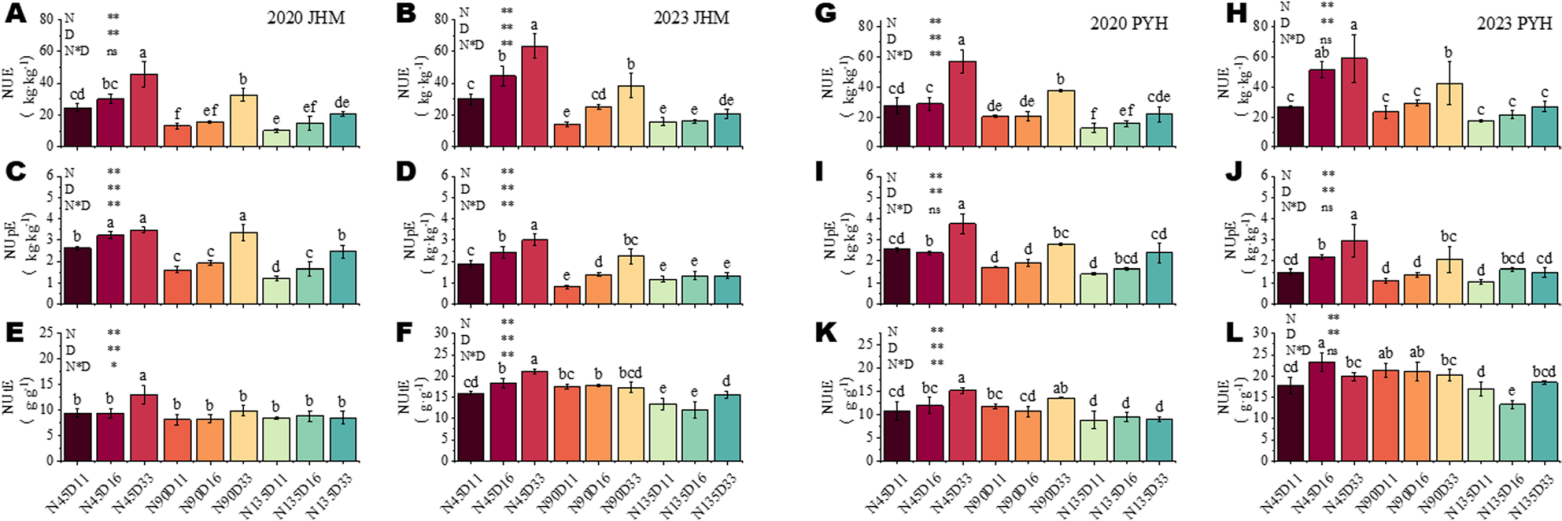
Interactive effects of nitrogen dose and planting density on NUE, NUpE and NUtE. **A**-**B** NUE of variety JHM in 2020 and 2023, respectively. **C**-**D** NUpE of variety JHM in 2020 and 2023, respectively. **E**-**F** NUtE of variety JHM in 2020 and 2023, respectively. **G**-**H** NUE of variety PYH in 2020 and 2023, respectively. **I**-**J** NUpE of variety PYH in 2020 and 2023, respectively. **K**-**L** NUtE of variety PYH in 2020 and 2023, respectively. Note: N45, N90, and N135 represent the nitrogen application rate of 45, 90, and 135 kg · ha^− 1^, respectively. D11, D16, and D33, and D33 represent planting density of 110,000, 160,000, and 330,000 plants per ha^− 1^, respectively. S1 represents the initial flowering period, three days after topdressing; S2 represents 20 days after topdressing; S3 represents 40 days after topdressing; and S4 represents 60 days after topdressing

High NUE of crops is essential to ensuring sustainable and risk-free production. The effects of nitrogen fertilizer and density on sesame NUE in 2020 and 2023 were similar (Fig. 4). For both JHM and PYH, NUE was highest under N45D33, with an averages of 54.4 g·g^− 1^ and 58.1 g·g^− 1^, respectively, followed by N90D33 (Fig. 4A, B,G, H). In both years, NUE and NUpE decreased with increasing N rate, but increased with higher density (Fig. 4A-D and G-J). The NUtE of JHM was the highest under N45D33 (average of 17.0 g·g^− 1^) (Fig. 4E, F). For PYH, it was highest under N45D33 (average of 15.2 g·g^− 1^) in 2020 and and under N45D16 (average of 23.29 g·g^− 1^) in 2023(Fig. 4K, L). The highest yield was achieved under N90D33, while the highest NUE was reached under N45D33, followed by N90D33. These results suggest that N45D33 optimized NUE by minimizing N input, but N90D33 balanced N supply and population yield potential. Taken together, these results indicate that moderate N input of ≤ 90 kg ha⁻¹ coupled with dense planting of 330,000 plants per hectare is suitable for higher yield in sesame. This optimal planting density and N rate may likely reduce environmental risk compared with high-N regimes. However, JHM and PYH showed differential responses to the interactive effects of planting density and N rate. For instance, in 2023, NUE of JHM was 6.78% higher than that of PYH under N45D33, while we observed the opposite trend under N90D33, with NUE of PYH 10.48% higher than that of JHM. Additional studies should investigate many other sesame varieties under varying environmental conditions to identify the most suitable planting density and N rate for sustainable enhanced production.

### *AMT1 *and *NRT2.13 A *regulated N metabolism to improve sesame NUE under optimal planting density and N rate

**Fig. 5:**
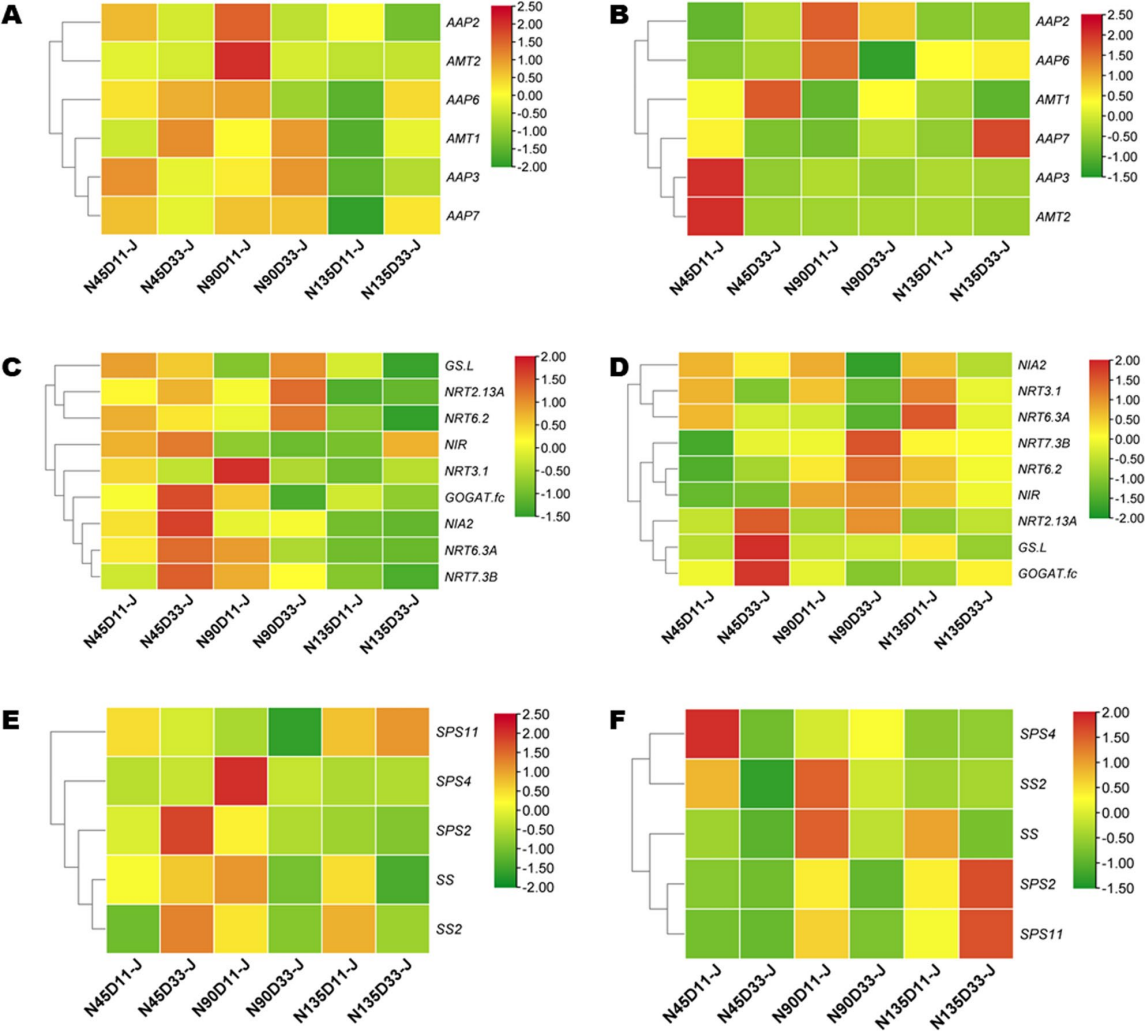
Interactive effects of nitrogen dose and planting density on the relative expression of N metabolism and sucrose metabolism-related genes in leaves of variety JHM in 2023. **A**-**B** Ammonium transporters at S2 and S3, respectively. **C**-**D** Nitrate transporters at S2 and S3, respectively. **E**-**F** Sucrose metabolism-related genes at S2 and S3, respectively. Note: N45, N90, and N135 represent the nitrogen application rate of 45, 90, and 135 kg · ha^− 1^, respectively. D11, D16, and D33, and D33 represent planting density of 110,000, 160,000, and 330,000 plants per ha^− 1^, respectively. S1 represents the initial flowering period, three days after topdressing; S2 represents 20 days after topdressing; S3 represents 40 days after topdressing; and S4 represents 60 days after topdressing

Tissue-specific nitrate transporters, ammonium transporters, or amino acid permeases, and GS strictly coordinate nitrogen metabolism in plants [39, 40]. Some nitrogen transporters are also regulated by carbon assimilators [41]. To gain insight into the mechanism underlying higher NUE under the dual influence of planting density and N rate, we examined the expression levels of nitrate transporters (NRT), ammonium transporters (AMT), amino acid permeases (AAP), glutamine synthetase (*GS*), and sucrose metabolism-related genes (sucrose synthase, SS and sucrose-phosphate synthase, SPS) in leaves at the S2 and S3 stages. The results showed that these genes are stage-specifically regulated, with their expression levels varying depending on the variety (Fig. 5A-F and S4A-F). *AMT1* was significantly induced in JHM under N45D33 and N90D33 at S2 and S3 (Fig. 5A, B). In PYH, *AMT1* expression was moderately high, while *AMT2* exhibited significantly high expression under N45D33 at S2 and under N90D33 at S3 (Figure S4A, B). *NRT2.13 A* showed significantly high expression levels in both JHM and PYH under N45D33 and N90D33 at the S2 and S3 stages (Fig. 5C, D and S4C, D). Correlation analysis revealed that NUE and NUpE were significantly positively correlated with *AMT1* (*r* = 0.96**) and *NRT2.13 A* (*r* = 0.93**) (Figure S4A, B). *AMT1* and *NRT2.13 A* showed a significant positive correlation, suggesting that they might interact to modulate nitrogen metabolism under optimum density and N rate conditions. These results indicate that *AMT1* and *NRT2.13 A* are strong candidates for regulating NUE under moderate N input and high planting density. A previous study in maize identified *AMT1;1b* as the key modulator of N metabolism and transfer among organs [20]. In JHM, *NIA2*, *NRT6.2*,* NRT7.3B*, and *GS.L* were significantly induced under N45D33 and N90D33 at S2 (Fig. 5C). *GOGAT.f c* was significantly induced under N45D33 at S2 and S3 (Fig. 5C, D). These genes may have also contributed to the high NUE recorded under N45D33 and N90D33. A previous study has speculated the importance of NRTs, GS, GOGAT, and GDH in mediating higher NUE in sesame [42]. A coordinated carbon and N metabolism is key to plant productivity and the maintaining of agroecosystem stability [36]. Regarding sucrose metabolism-related genes, *SPS2* and *SS2* were significantly induced under N45D33 at S2 in JHM (Fig. 5E). In PYH, *SPS4* was highly induced under N45D33 at S2 and under N90D33 at S3 (Figure S4E, F). *SS* was highly induced under N90D33 at S3 in PYH (Fig. 5E). These genes might also participate in regulating N metabolism in sesame under the dual influence of planting density and N rate. Functional characterization (e.g., transgenic, mutant, or association studies) of identified genes, notably *AMT1* and *NRT2.13 A* in sesame N metabolism, is required to reveal their specific regulatory functions and to dissect the molecular network that controls sesame NUE and yield under moderate N input and dense planting conditions.

**Fig. 6:**
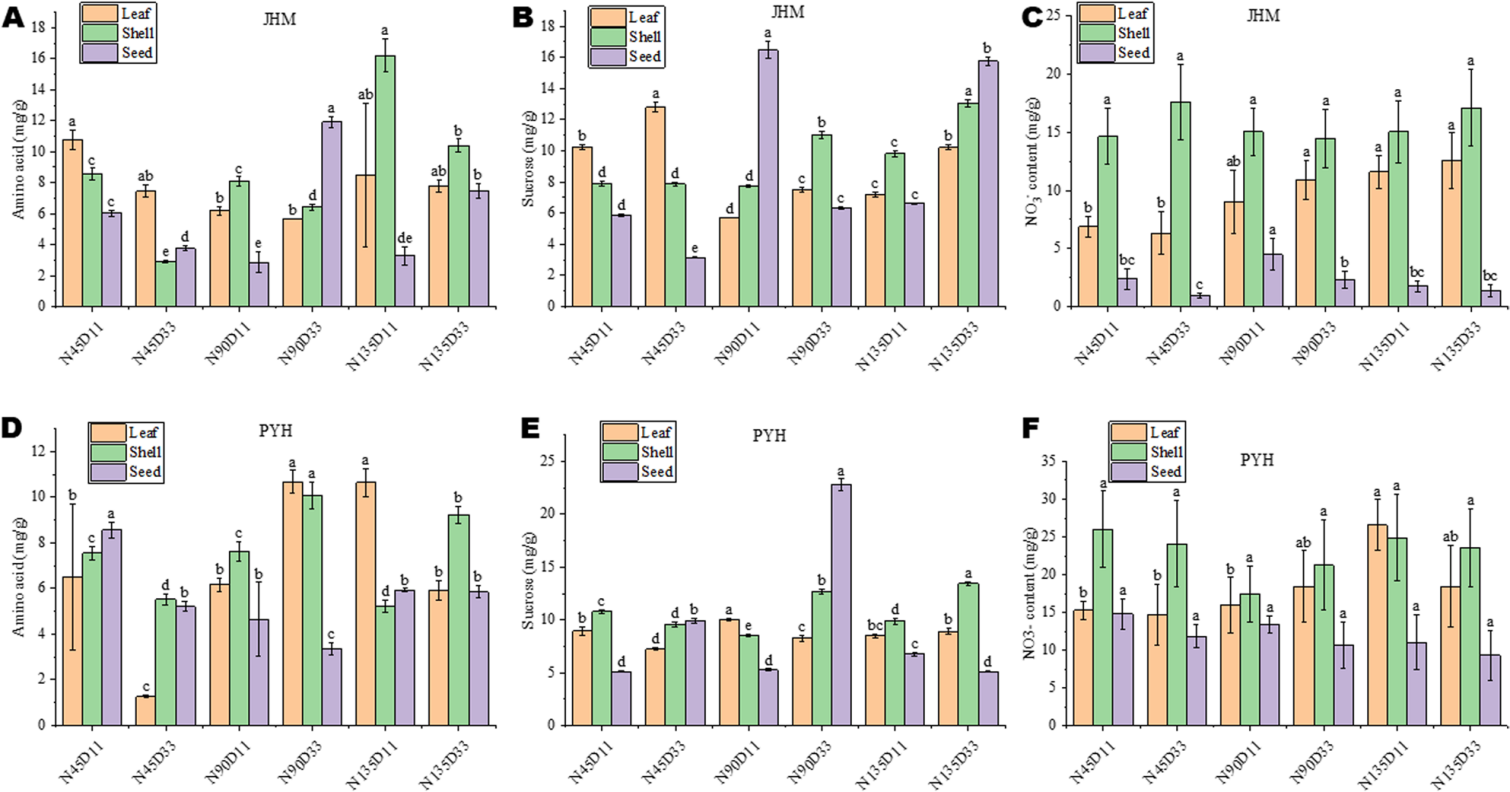
Interactive effects of nitrogen dose and planting density on the content of amino acids, sucrose, and nitrate in different sesame tissues at the maturity stage. **A**-**C** Amino acids, sucrose, and nitrate contents of different tissues of variety JHM. **D**-**F** Amino acids, sucrose, and nitrate contents of different tissues of variety PYH. Note: N45, N90, and N135 represent the nitrogen application rate of 45, 90, and 135 kg · ha^− 1^, respectively. D11, D16, and D33, and D33 represent planting density of 110,000, 160,000, and 330,000 plants per ha^− 1^, respectively. S1 represents the initial flowering period, three days after topdressing; S2 represents 20 days after topdressing; S3 represents 40 days after topdressing; and S4 represents 60 days after topdressing

In addition to the expression levels of key genes, we assessed the nitrate, amino acid, and sucrose contents in different tissues of sesame. The results also showed varietal-specific responses to planting density and N rate. There was no significant difference in seed nitrate content among treatments in PYH (Fig. 6F). In contrast, seed nitrate content under N90D11 was the highest (Fig. 6E). Under N90D33, seed amino acid content was the highest in JHM, while sucrose content was the highest in PYH (Fig. 6A, E). These results indicate that amino acid content in sesame varieties could be improved by balancing planting density and N rate. A deep analysis of nitrogen and carbon metabolism in JHM and PYH under N90D33 may identify key genetic resources for quality breeding in sesame.

### Moderate N input coupled with dense planting improved sesame OC and UFA content

Oil content (OC), protein content (PC), and FA composition are critical quality traits in sesame [43]. The PC, OC, and FA profiles of black sesame seeds under different planting densities and N doses in 2020 and 2023 are presented in Table 1 and Table S6, respectively. Under the same N application rate, PC decreased with increasing density, while OC increased with density. For instance, from N90D11 to N90D33 in 2020, PC content in JHM was reduced by 2.18%, while OC was increased by 3.05% (Table 1). At the meantime in PYH, PC content was reduced by 10.04%, while OC was increased by 2.78% (Table 1). These results further support the conclusion of varietal-specific responses to the interactive effects of planting density and N rate in sesame. These results suggest that planting density and N rate may alter the allocation of intermediate metabolic resources required for the synthesis of oil bodies. As a support, a study revealed that changing N rate and planting density modify the supply of free amino acid to protein synthesis and alter the expression of the storage protein gene in soft wheat [44]. In contrast to PC and OC, FA composition fluctuated, mainly revealing a significant influence of planting density, N rate, variety, and growing conditions. For instance, no significant differences were observed between oleic acid and linoleic acid content among treatments in JHM in 2020, whereas significant differences were observed in 2023 (Table 1 and Table S6). Similarly, no significant differences were observed between oleic acid content among treatments in PYH in 2023, whereas significant differences were observed in 2020 (Table 1 and Table S6). These results emphasize the complexity of FA acid regulation mechanisms in sesame, which is governed by complex genetic and environmental factors [45–47]. The highest OC (52.27% and 54.6% for JHM and PYH, respectively, in 2023) and UFA (87.94% and 86.54% for JHM and PYH, respectively, in 2023) content were obtained under N45D33 for the two varieties (Table 1 and S6). It was followed by N90D33, with and OC of 50.06% and 53.26% for JHM and PYH, respectively, and UFA of 85.88% and 86.24% for JHM and PYH, respectively, also in 2023 (Table 1 and S6). Collectively, our results indicate varietal-specific responses to the interactive effects of planting density and N rate in sesame. Furthermore, they show that moderate N input of ≤ 90 kg ha⁻¹ coupled with dense planting of 330,000 plants per hectare not only improved sesame growth, NUE, and yield, but also key quality traits, including OC and UFA content. The enhancement of OC and UFA composition under the optimal condition of N90D33 may imply a reduced N competition, which diverts carbon metabolites from protein synthesis to oil biosynthesis.

**Table 1:** Interactive effects of nitrogen dose and planting density on seed oil content, protein content, and fatty acid composition of JHM and PYH in the year 2020

Variety	Treatment	Fat	Protein	SFA	UFA	oleic acid	linoleic acid	palmitic acid	stearic acid
JHM	N45D11	49.18 ± 0.32 ab	25.42 ± 0.42 abc	15.05 ± 0.06 a	84.8 ± 0.64 a	35.23 ± 2.27 a	48.91 ± 2.9 a	8.98 ± 0.04 bc	5.19 ± 0.06 a
	N45D16	48.7 ± 1.02 bc	24.44 ± 1.11 cd	14.79 ± 0.2 ab	85.26 ± 0.55 a	34.31 ± 1.81 a	50.28 ± 2.29 a	8.98 ± 0.04 bc	4.96 ± 0.21 ab
	N45D33	49.93 ± 0.22 a	24.12 ± 0.65 d	14.77 ± 0.05 ab	85.27 ± 0.08 a	34.48 ± 0.35 a	50.14 ± 0.29 a	8.98 ± 0.01 bc	4.93 ± 0.03 ab
	N90D11	48.7 ± 0.55 bc	25.14 ± 0.23 bc	14.76 ± 0.2 ab	85.1 ± 0.24 a	33.55 ± 0.21 a	50.87 ± 0.21 a	8.97 ± 0.03 bc	4.94 ± 0.22 ab
	N90D16	48.41 ± 0.37 bc	25.18 ± 0.43 bc	14.87 ± 0.2 ab	84.84 ± 0.53 a	33.95 ± 1.65 a	50.23 ± 2.05 a	8.97 ± 0.02 bc	5.03 ± 0.2 ab
	N90D33	50.19 ± 0.94 a	24.59 ± 0.36 cd	14.73 ± 0.27 ab	85.29 ± 0.68 a	34.9 ± 0.6 a	49.72 ± 0.09 a	8.96 ± 0.01 bc	4.94 ± 0.27 ab
	N135D11	47.05 ± 0.99 d	26.29 ± 0.41 a	14.7 ± 0.22 b	85.03 ± 0.35 a	33.73 ± 1.11 a	50.59 ± 0.95 a	9.03 ± 0.02 ab	4.81 ± 0.22 b
	N135D16	47.95 ± 0.39 cd	25.73 ± 0.46 ab	14.9 ± 0.12 ab	85.04 ± 0.14 a	34.04 ± 0.6 a	50.33 ± 0.53 a	9 ± 0.03 a	5.02 ± 0.14 ab
	N135D33	49.35 ± 0.76 ab	25.09 ± 0.38 bc	14.97 ± 0.3 ab	84.49 ± 0.73 a	33.37 ± 1.24 a	50.44 ± 1.94 a	8.94 ± 0.04 c	5.19 ± 0.35 a
	N	**	**	ns	ns	ns	ns	ns	ns
	D	**	**	ns	ns	ns	ns	*	ns
	N*D	ns	ns	ns	ns	ns	ns	*	ns
PYH	N45D11	50.97 ± 1.2 bc	22.63 ± 0.43 cd	15.01 ± 0.25 abc	85.12 ± 0.98 c	36.9 ± 0.96 a	47.57 ± 1.91 b	8.97 ± 0.02 ab	5.21 ± 0.22 ab
	N45D16	51.44 ± 0.33 abc	22.75 ± 1.11 bcd	14.97 ± 0.16 abc	85.76 ± 0.09 ab	36.07 ± 0.64 a	49.04 ± 0.61 ab	8.96 ± 0.01 ab	5.17 ± 0.15 ab
	N45D33	52.07 ± 0.24 ab	22.19 ± 0.52 d	14.83 ± 0.14 bc	85.97 ± 0.08 a	36.52 ± 1.02 a	48.79 ± 0.95 ab	8.97 ± 0.01 ab	5.03 ± 0.14 ab
	N90D11	51.02 ± 0.66 bc	22.7 ± 0.52 bcd	14.81 ± 0.14 bc	85.5 ± 0.24 abc	35.17 ± 0.58 a	49.67 ± 0.64 ab	8.99 ± 0.03 a	4.98 ± 0.14 ab
	N90D16	50.84 ± 0.94 bc	22.85 ± 1.25 bcd	14.9 ± 0.42 abc	85.21 ± 0.1 bc	35.32 ± 2.4 a	49.22 ± 2.48 ab	8.99 ± 0.02 a	5.07 ± 0.41 ab
	N90D33	52.44 ± 0.78 a	20.42 ± 0.3 e	14.68 ± 0.13 c	86.01 ± 0.15 a	34.91 ± 1.22 a	50.44 ± 1.18 a	8.97 ± 0.01 ab	4.89 ± 0.13 b
	N135D11	48.51 ± 0.53 d	25.59 ± 0.03 a	15.22 ± 0.05 a	84.41 ± 0.29 d	36.31 ± 0.41 a	47.46 ± 0.65 b	8.99 ± 0.03 a	5.33 ± 0.07 a
	N135D16	50.36 ± 0.76 c	23.68 ± 0.17 b	14.81 ± 0.27 bc	85.15 ± 0.36 c	35.49 ± 1.92 a	48.99 ± 2 ab	8.97 ± 0.02 ab	5.01 ± 0.27 ab
	N135D33	51.57 ± 1.17 abc	23.46 ± 0.14 ab	15.1 ± 0.23 ab	85.13 ± 0.41 c	36.3 ± 0.38 a	48.18 ± 0.71 b	8.95 ± 0.02 b	5.3 ± 0.25 a
	N	**	**	*	**	*	*	ns	*
	D	**	**	ns	**	ns	ns	ns	ns
	N*D	*	**	ns	ns	ns	ns	ns	ns

N45, N90, and N135 represent the nitrogen application rate of 45, 90, and 135 kg · ha− 1, respectively. D11, D16, and D33, and D33 represent planting density of 110000, 160000, and 330000 plants per ha− 1, respectively. S1 represents the initial flowering period, three days after topdressing; S2 represents 20 days after topdressing; S3 represents 40 days after topdressing; and S4 represents 60 days after topdressing

Significant interannual differences were observed for most measured traits, indicating that sesame agronomic traits are sensitive to fluctuations in environmental conditions. Supporting this, temperature and precipitation differed between the growing periods in 2020 and 2023 (Figure S1). Adopting these optimal planting conditions in the future, after a comprehensive evaluation of large sesame germplasm across varying environmental conditions, may substantially reduce production costs for farmers, thereby increasing seed market value and incomes. In-depth molecular investigations of developing sesame plants under these optimal conditions are also needed to elucidate the mechanisms underlying these observed improvements.

## Conclusions

Overall, this study comprehensively revealed that sesame’s response to the dual effects of planting density and N application rate is developmentally regulated and varietal specific. Notably, we found that moderate nitrogen application of ≤ 90 kg ha⁻¹ coupled with dense planting of ⁓330,000 plants per hectare improved sesame growth, NUE, yield, and seed quality. Under this optimal condition, yield and seed oil and UFA contents reached their maximum in both JHM and PYH. The number of capsules in PYH was hardly affected by density, making it more suitable for dense planting than JHM. *AMT1* and *NRT2.13 A* were identified as key regulatory genes of sesame NUE and modulators of N metabolism under optimal planting density and N rate. Other candidate regulatory genes, including *NIA2*, *NRT6.2*,* NRT7.3B*,* GS.L* and *GOGAT.fc* were also identified. Sucrose metabolism-related genes, *SPS2*, *SS2*, *SPS4*, and *SS*, may also influence N metabolism in sesame under different planting densities and N rates, highlighting the need to dissect interactions between N metabolism and carbon metabolism in sesame. Our findings provide strong evidence for improved yield, NUE, and selected seed-quality traits under moderate N input and high density in the studied environment, and frame broader sustainability implications as hypotheses or future directions rather than definitive conclusions. Moreover, they offer important resources for dissecting the molecular regulation of N metabolism under moderate N input and dense planting.

## Materials and methods

### Site description

Field experiments were conducted at the Jiangxi Agriculture University (28.76 N, 115.86 E, 20 m above sea level), Jiangxi Province, China, in 2020 and 2023. The site is located in a subtropical monsoon climate zone. The temperature, rainfall and annual sunshine are shown in Supplementary Fig. 1. The physicochemical properties of the soil are shown in Supplementary Table 1.

### Experiment design

‘Jinhuangma’ (JHM) and ‘Poyanghei’ (PYH), two sesame varieties widely cultivated in Jiangxi Province, were used in this study. The two varieties were grown under a combination of different planting densities and N rates, including N45D11, N45D16, N45D33, N90D11, N90D16, N90D33, N135D11, N135D16, and N135D33,. D11, D16, and D33 indicate planting density of 110,000, 160,000, and 330,000 plants per hectare, respectively. N45, N90, and N135 indicate nitrogen (urea) application rates of 45 kg, 90 kg, and 135 kg of N per hectare, respectively. Plot size was 16 m^2^ (4 m × 4 m), and the plots were arranged in a completely randomized design with three replications. Definitions of the growth periods are presented in Supplementary Table 2. When 2–3 pairs of leaves grew, the seedlings were thinned according to the corresponding density. Phosphate (P₂O₅) and potassium (K₂O) fertilizers were applied (sprinkled before sowing) once as a basal at the rate of 50 kg·ha^− 1^ and 100 kg·ha^− 1^, respectively.

### Biomass, grain yield, and yield components

Phenotypic traits were evaluated following a previously described method [48]. For the aboveground biomass, samples were taken at the beginning of flowering (3 days after top dressing), full flowering (20 days after top dressing), tail flowering (40 days after top dressing), and maturity (harvest). Three representative plants were taken from each plot. After the plant height was measured, stems and leaves were separated and oven-dried at 105 ℃ for 30 min, followed by drying at 75 °C until a constant weight was achieved. The leaf area index (LAI) was determined by the specific leaf weight method.

Uniformly growing plants were sampled from each plot at the seed physiological maturity stage to evaluate yield and its components. The number of initial capsules, the number of single capsules, the fruit axis length, the capsule number per plant, and the fruit node density were quantified. Ten fruits were taken and put in an envelope bag, naturally dried in the sun, followed by the determination of the number of seeds per capsule, seed weight, 1000-seeds weight, and capsule weight.

### Evaluation of total nitrogen content and NUE

Four representative plants per treatment were harvested at different growth stages to evaluate the total nitrogen (TN) contents. Samples of each plant part were ground separately and passed through a 0.2 mm sieve. TN content was measured by the Kjeldahl nitrogen meter [49]. Nitrogen accumulation and NUE were calculated using:

$$\begin{aligned}&\mathrm{Nitrogen}\;\mathrm{use}\;\mathrm{efficiency}\;(\mathrm{NUE},\;\%)\;\\&=\;(\mathrm{grain}\;\mathrm{yield}/\;\mathrm{applied}\;\mathrm N)\;\times\;100\end{aligned}$$

$$\begin{aligned}&\mathrm{Nitrogen}\;\mathrm{utilization}\;\mathrm{efficiency}\;(\mathrm{NUtE},\;\mathrm g\;\mathrm g^{-1})\;\\&=\;\mathrm{grain}\;\mathrm{weight}/\mathrm{total}\;\mathrm{nitrogen}\;\mathrm{per}\;\mathrm{plant}\end{aligned}$$

$$\begin{aligned}&\mathrm{Nitrogen}\;\mathrm{uptake}\;\mathrm{efficiency}\;(\mathrm{NUpE},\;\mathrm{kg}\;\mathrm{kg}^{-1})\;\\&=\;\mathrm{TN}\;\mathrm{per}\;\mathrm{unit}/\;\mathrm N\;\mathrm{application}\;\mathrm{per}\;\mathrm{unit}\;\times\;100\end{aligned}$$

### SPAD and photosynthetic rate

At the initial-flowering stage (PF), mid-flowering stage (MF), late-flowering stage (LF) and maturity stage (FM), SPAD values were measured on the 30 uppermost fully expanded leaves per plot by using a chlorophyll meter [SPAD-502, Soil Plant Analysis Development (SPAD) Section, Minolta Camera Co., Osaka, Japan]. Fifteen fully expanded 4th leaves (from top) were chosen for the determination of photosynthetic rate (Pn).

### Evaluation of nitrate (NO_3_^−^) concentration

The NO3- content was evaluated via the nitration of salicylic acid method by using a UV spectrophotometer (Youke UV-722; Youke, Corporation, Shanghai, China) at 410 nm [50, 51]. Samples were ground and sieved (0.25 mm). Then, an accurate 0.1 g of sample was extracted with 5 mL of water in a 45 ℃ water bath for 1 h, followed by centrifuging at 5,000 g for 5 min. The reaction mixture contained 50 µL supernatant and 200 µL sulfosalicylic acid. After reacting for 20 min, 4.75 mL of 8 M NaOH was immediately added, and the absorbance was recorded at 410 nm.

### RT-qPCR

Leaf samples were collected during the tail flowering period and stored at −80 °C in an ultra-low temperature refrigerator. Total RNA was extracted using an RNA extraction kit (Takara, Code 9769 S) and reverse transcription (RT) was performed using PrimeScriptTM 2II.1st Strand cDNA Synthesis Kit (Takara, Code 6210 A). qPCR amplification was performed using TB Green^®^ *Premix Ex Taq*™ II (Takara, Code No. RR820A). β-Actin (ncbi_105159390) was used as an internal control to normalize the expression levels of target genes [52]. The primers were designed using the primer design program of Jinruisi Biotechnology, and are listed in the Supplementary Table 3.

### Oil content, fatty acid composition, and protein content

Oils, fatty acid composition, and protein content were determined using near-infrared spectroscopy [53]. The seeds were harvested at maturity, and samples were detected by a DS2500F-type near-infrared spectrometer (Foss, USA) and repeated three times.

### Data analysis

All experiments were conducted with three replicates. Data variance analysis was performed using SPSS 24.0. Significant differences in yield were observed both between the two years and among different varieties within the same year. Subsequently, the significances and main effects between treatments (nitrogen × density) were tested separately for each year and variety. For all significance analyses, one-way ANOVA was used, and Duncan’s multiple range test was applied for post hoc multiple comparisons (*P* ˂ 0.05).

## Supplementary Information

Supplementary Material 1.

Supplementary Material 2.

## Data Availability

The data that support the findings of this study are available from the corresponding author upon reasonable request.

## References

[1] Segla Koffi Dossou S, Xu F, You J, Zhou R, Li D, Wang L. Widely targeted metabolome profiling of different colored Sesame (Sesamum indicum L.) seeds provides new insight into their antioxidant activities. Food Res Int. 2022;151:110850. 34980388 10.1016/j.foodres.2021.110850

[2] Xu Z, Li M, Jiang N, Gui C, Wang Y, An Y, Xiang X, Deng Q. Black Sesame seeds: nutritional value, health benefits, and food industrial applications. Trends Food Sci Technol. 2024;153:104740.

[3] Wang R, Xiao Y, Lv F, Hu L, Wei L, Yuan Z, et al. Bacterial community structure and functional potential of rhizosphere soils as influenced by nitrogen addition and bacterial wilt disease under continuous sesame cropping. Appl Soil Ecol. 2018;125:117–27.

[4] Zenawi G, Mizan A. Effect of nitrogen fertilization on the growth and seed yield of Sesame (Sesamum indicum L). Int J Agron. 2019;2019:1–7.

[5] Myint D, Gilani SA, Kawase M, Watanabe KN. Sustainable sesame (*Sesamum indicum* L.) production through improved technology: an overview of production, challenges, and opportunities in Myanmar. Sustainability. 2020;12(9):3515.

[6] Yadav R, Kalia S, Rangan P, Pradheep K, Rao GP, Kaur V, Pandey R, Rai V, Vasimalla CC, Langyan S, et al. Current research trends and prospects for yield and quality improvement in Sesame, an important oilseed crop. Front Plant Sci. 2022;13:863521. 35599863 10.3389/fpls.2022.863521 PMC9120847

[7] Rathore VS, Nathawat NS, Meel B, Bhardwaj S. Cultivars and nitrogen application rates affect yield and nitrogen use efficiency of wheat in hot arid region. Proceedings of the National Academy of Sciences, India Section B: Biological Sciences. 2017;87(4):1479–88.

[8] Cong XH, Shi FZ, Ruan XM, Luo YX, Ma TC, Luo ZX. [Effects of nitrogen fertilizer application rate on nitrogen use efficiency and grain yield and quality of different rice varieties]. Ying Yong Sheng Tai Xue Bao. 2017;28(4):1219–26. 29741319 10.13287/j.1001-9332.201704.010

[9] Stockmann F, Weber EA, Merkt N, Schreiter P, Claupein W, Graeff-Hoenninger S. Impact of row distance and seed density on grain yield, quality traits, and free asparagine of organically grown wheat. Agronomy-Basel. 2019;9(11):713.

[10] Huang M, Chen J, Cao F, Zou Y. Increased hill density can compensate for yield loss from reduced nitrogen input in machine-transplanted double-cropped rice. Field Crops Res. 2018;221:333–8.

[11] Hou W, Khan MR, Zhang J, Lu J, Ren T, Cong R, Li X. Nitrogen rate and plant density interaction enhances radiation interception, yield and nitrogen use efficiency of mechanically transplanted rice. Agric Ecosyst Environ. 2019;269:183–92.

[12] Shi D-y, Li Y-h, Zhang J-w, Liu P, Zhao B, Dong S. -t: increased plant density and reduced N rate lead to more grain yield and higher resource utilization in summer maize. J Integr Agric. 2016;15(11):2515–28.

[13] Zheng B, Zhang X, Wang Q, Li W, Huang M, Zhou Q, Cai J, Wang X, Cao W, Dai T, et al. Increasing plant density improves grain yield, protein quality and nitrogen agronomic efficiency of soft wheat cultivars with reduced nitrogen rate. Field Crops Res. 2021;267:108145.

[14] Khan S, Anwar S, Kuai J, Ullah S, Fahad S, Zhou G. Optimization of nitrogen rate and planting density for improving Yield, nitrogen use Efficiency, and lodging resistance in oilseed rape. Front Plant Sci. 2017;8:532. 10.3389/fpls.2017.00532 PMC5423294 28536581

[15] Lin S-H, Kuo H-F, Gv C, Lin C-S, Lepetit M, Hsu P-K, Tillard P, Lin H-L, Wang Y-Y, Tsai C-B, et al. Mutation of the Arabidopsis NRT1.5 nitrate transporter causes defective Root-to-Shoot nitrate transport. Plant Cell. 2008;20(9):2514–28. 18780802 10.1105/tpc.108.060244 PMC2570733

[16] Giehl RFH, Laginha AM, Duan F, Rentsch D, Yuan L. Von Wirén N: A critical role of AMT2;1 in Root-To-Shoot translocation of ammonium in Arabidopsis. Mol Plant. 2017;10(11):1449–60. 29032248 10.1016/j.molp.2017.10.001

[17] Tegeder M, Masclaux-Daubresse C. Source and sink mechanisms of nitrogen transport and use. New Phytol. 2018;217(1):35–53. 29120059 10.1111/nph.14876

[18] Wang Y, Fu B, Pan L, Chen L, Fu X, Li K. Overexpression of ArabidopsisDof1, GS1 and GS2 enhanced nitrogen assimilation in transgenic tobacco grown under low-nitrogen conditions. Plant Mol Biol Rep. 2013;31(4):886–900.

[19] Zhang Z, Hu B, Chu C. Towards understanding the hierarchical nitrogen signalling network in plants. Curr Opin Plant Biol. 2020;55:60–5. 32304938 10.1016/j.pbi.2020.03.006

[20] Duan F, Wei Z, Soualiou S, Zhou W. Nitrogen partitioning in maize organs and underlined mechanisms from different plant density levels and N application rate in China. Field Crops Res. 2023;294:108874.

[21] Artins A, Martins MCM, Meyer C, Fernie AR, Caldana C. Sensing and regulation of C and N metabolism – novel features and mechanisms of the TOR and SnRK1 signaling pathways. Plant J. 2024;118(5):1268–80. 38349940 10.1111/tpj.16684

[22] Kusano M, Fukushima A, Redestig H, Saito K. Metabolomic approaches toward understanding nitrogen metabolism in plants. J Exp Bot. 2011;62(4):1439–53. 21220784 10.1093/jxb/erq417

[23] Wu X, Tong L, Kang S, Du T, Ding R, Li S, Chen Y. Combination of suitable planting density and nitrogen rate for high yield maize and their source–sink relationship in Northwest China. J Sci Food Agric. 2023;103(11):5300–11. 37016583 10.1002/jsfa.12602

[24] Tian LB, Shen ZY, Zhao XT, Zhang F, Hou WF, Gao Q, et al. Interactive effects of planting density and nitrogen application rate on plant grain yield and water use efficiency of two maize cultivars. Sci Agric Sin. 2024;57(21):4221–37.

[25] Wu Q, Zhang Y, Zhao Z, Xie M, Hou D. Estimation of relative chlorophyll content in spring wheat based on multi-temporal UAV remote sensing. Agronomy. 2023;13(1):211.

[26] Li G, Pan J, Cui K, Yuan M, Hu Q, Wang W, Mohapatra PK, Nie L, Huang J, Peng S. Limitation of unloading in the developing grains is a possible cause responsible for low stem Non-structural carbohydrate translocation and poor grain yield formation in rice through verification of Recombinant inbred lines. Front Plant Sci. 2017;8:1369. 28848573 10.3389/fpls.2017.01369 PMC5550689

[27] Guo J, Li H, Zhou C, Yang Y. Effects of flag leaf and number of vegetative Ramets on sexual reproductive performance in the clonal grass Leymus chinensis. Front Plant Sci. 2020;11:534278. 33193474 10.3389/fpls.2020.534278 PMC7661390

[28] Zhang Y, Xu Z, Li J, Wang R. Optimum planting density improves resource use efficiency and yield stability of rainfed maize in semiarid climate. Front Plant Sci. 2021;12:752606. 34868140 10.3389/fpls.2021.752606 PMC8633400

[29] Luo C, Guo Z, Xiao J, Dong K, Dong Y. Effects of applied ratio of nitrogen on the light environment in the canopy and Growth, development and yield of wheat when intercropped. Front Plant Sci. 2021;12:719850. 34490016 10.3389/fpls.2021.719850 PMC8417318

[30] Zhou C, Jia B, Wang S, Huang Y, Wang Y, Han K, Wang W. Effects of nitrogen fertilizer applications on photosynthetic production and yield of Japonica rice. Int J Plant Prod. 2021;15(4):599–613.

[31] Xiong H, Wang R, Jia X, Sun H, Duan R. Transcriptomic analysis of rapeseed (Brassica napus. L.) seed development in Xiangride, Qinghai Plateau, reveals how its special eco-environment results in high yield in high-altitude areas. Front Plant Sci. 2022;13:927418. 35982704 10.3389/fpls.2022.927418 PMC9379305

[32] Zheng S, Ye C, Lu J, Liufu J, Lin L, Dong Z, Li J, Zhuang C. Improving the rice photosynthetic efficiency and yield by editing OsHXK1 via CRISPR/Cas9 system. Int J Mol Sci. 2021;22(17):9554. 10.3390/ijms22179554 PMC8430575 34502462

[33] Zhou R, Dossa K, Li D, Yu J, You J, Wei X, Zhang X. Genome-Wide association studies of 39 seed Yield-Related traits in Sesame (Sesamum indicum L). Int J Mol Sci. 2018;19(9):2794. 10.3390/ijms19092794 PMC6164633 30227628

[34] Alain G. Nitrogen nutrition in plants: rapid progress and new challenges. J Exp Bot. 2017;68(10):2457–62. 10.1093/jxb/erx171 PMC5853562 30053117

[35] Kun X, Yuhan R, Aiqun C, Congfan Y, Qingsong Z, Jun C, Dongsheng W, Yiting L, Shuijin H, Guohua X. Plant nitrogen nutrition: the roles of arbuscular mycorrhizal fungi. J Plant Physiol. 2022;269:153591. 10.1016/j.jplph.2021.153591 34936969

[36] Liu H, Gao X, Fan W, Fu X. Optimizing carbon and nitrogen metabolism in plants: from fundamental principles to practical applications. J Integr Plant Biol. 2025;67(6):1447–66. 40376749 10.1111/jipb.13919

[37] Liang C, Wang Y, Zhu Y, Tang J, Hu B, Liu L, Ou S, Wu H, Sun X, Chu J, et al. OsNAP connects abscisic acid and leaf senescence by fine-tuning abscisic acid biosynthesis and directly targeting senescence-associated genes in rice. Proc Natl Acad Sci U S A. 2014;111(27):10013–8. 24951508 10.1073/pnas.1321568111 PMC4103337

[38] Liu X, Liu HF, Li HL, An XH, Song LQ, You CX, Zhao LL, Tian Y, Wang XF. MdMYB10 affects nitrogen uptake and reallocation by regulating the nitrate transporter MdNRT2.4-1 in the red flesh Apple. Hortic Res. 2022;9:uhac016. 10.1093/hr/uhac016 PMC9016894 35184189

[39] Hamada M, Schröder K, Bathia J, Kürn U, Fraune S, Khalturina M, Khalturin K, Shinzato C, Satoh N, Bosch TC. Metabolic co-dependence drives the evolutionarily ancient Hydra-Chlorella symbiosis. Elife. 2018;7:e35122. 10.7554/eLife.35122 PMC6019070 29848439

[40] Xin W, Zhang L, Zhang W, Gao J, Yi J, Zhen X, Li Z, Zhao Y, Peng C, Zhao C. An integrated analysis of the rice transcriptome and metabolome reveals differential regulation of carbon and nitrogen metabolism in response to nitrogen availability. Int J Mol Sci. 2019;20(9):2349. 10.3390/ijms20092349 PMC6539487 31083591

[41] Zhao X, Sun XF, Zhao LL, Huang LJ, Wang PC. Morphological, transcriptomic and metabolomic analyses of sophora Davidii mutants for plant height. BMC Plant Biol. 2022;22(1):144. 35337273 10.1186/s12870-022-03503-1 PMC8951708

[42] Wang M, Wang Y, Wang X, Wei G, Yang H, Yang X, Shen T, Qu H, Fang S, Wu Z. Integrated physiological, biochemical, and transcriptomics analyses reveal the underlying mechanisms of high nitrogen use efficiency of black Sesame. Plant Physiol Biochemistry: PPB. 2024;206:108205. 10.1016/j.plaphy.2023.108205 38035467

[43] Wei P, Zhao F, Wang Z, Wang Q, Chai X, Hou G, et al. Sesame (*Sesamum indicum* L.): a comprehensive review of nutritional value, phytochemical composition, health benefits, development of food, and industrial applications. Nutrients. 2022. 10.3390/nu14194079. 36235731 10.3390/nu14194079 PMC9573514

[44] Zheng B, Jiang J, Wang L, Huang M, Zhou Q, Cai J, Wang X, Dai T, Jiang D. Reducing nitrogen rate and increasing plant density accomplished high yields with satisfied grain quality of soft wheat via modifying the free amino acid supply and storage protein gene expression. J Agric Food Chem. 2022;70(7):2146–59. 35142500 10.1021/acs.jafc.1c07033

[45] Zhou W, Song S, Segla Koffi Dossou S, Zhou R, Wei X, Wang Z, et al. Genome-wide association analysis and transcriptome reveal novel loci and a candidate regulatory gene of fatty acid biosynthesis in sesame (*Sesamum indicum* L). Plant Physiol Biochem. 2022;186:220–31. 35921726 10.1016/j.plaphy.2022.07.023

[46] Bekele B, Andargie M, Gallach M, Beyene D, Tesfaye K. Decoding gene expression dynamics during seed development in Sesame (Sesamum indicum L.) through RNA-Seq analysis. Genomics. 2025;117(2):110997. 39809365 10.1016/j.ygeno.2025.110997

[47] Bhunia RK, Kaur R, Maiti MK. Metabolic engineering of fatty acid biosynthetic pathway in sesame (*Sesamum indicum* L.): assembling tools to develop nutritionally desirable sesame seed oil. Phytochem Rev. 2016;15(5):799–811.

[48] Yemata​ G, Bekele T. Evaluation of sesame (Sesamum indicum L.) varieties for drought tolerance using agromorphological traits and drought tolerance indices. PeerJ. 2024;12:e16840. 10.7717/peerj.16840 PMC10838076 38313022

[49] Singh P, Singh RK, Song Q-Q, Li H-B, Yang L-T, Li Y-R. Methods for Estimation of Nitrogen Components in Plants and Microorganisms. In: Gupta KJ, editor. Nitrogen Metabolism in Plants: Methods and Protocols. New York, NY: Springer New York; 2020. p. 103–12. 10.1007/978-1-4939-9790-9_10 31595474

[50] Cataldo DA, Maroon M, Schrader LE, Youngs VL. Rapid colorimetric determination of nitrate in Plant-Tissue by nitration of Salicylic-Acid. Commun Soil Sci Plant Anal. 1975;6(1):71–80.

[51] Gao K, Chen F, Yuan L, Zhang F, Mi G. A comprehensive analysis of root morphological changes and nitrogen allocation in maize in response to low nitrogen stress. Plant Cell Environ. 2015;38(4):740–50. 25159094 10.1111/pce.12439

[52] Li D, Liu P, Yu J, Wang L, Dossa K, Zhang Y, et al. Genome-wide analysis of WRKY gene family in the sesame genome and identification of the WRKY genes involved in responses to abiotic stresses. BMC Plant Biol. 2017;17(1):152. 28893196 10.1186/s12870-017-1099-y PMC5594535

[53] Liu Y-y, Mei H-x, Du Z-w, Wu K, Zhang H-y. Zheng Y-z: nondestructive Estimation of fat constituents of Sesame (Sesamum indicum L.) seeds by Near-Infrared reflectance spectroscopy. J Am Oil Chem Soc. 2015;92(7):1035–41.

