# Supplementary material for: Physiological and molecular insights into nitrogen rate and planting density interactive regulation of black sesame nitrogen use efficiency, growth, yield, and seed quality
Source: BMC Plant Biol. 2026 Jan 22;26:317. doi: 10.1186/s12870-026-08114-8 (PMC12911174; doi:10.1186/s12870-026-08114-8)
Supplement: Supplementary file 2 — Supplementary Material 2. [file 12870_2026_8114_MOESM2_ESM.pdf]

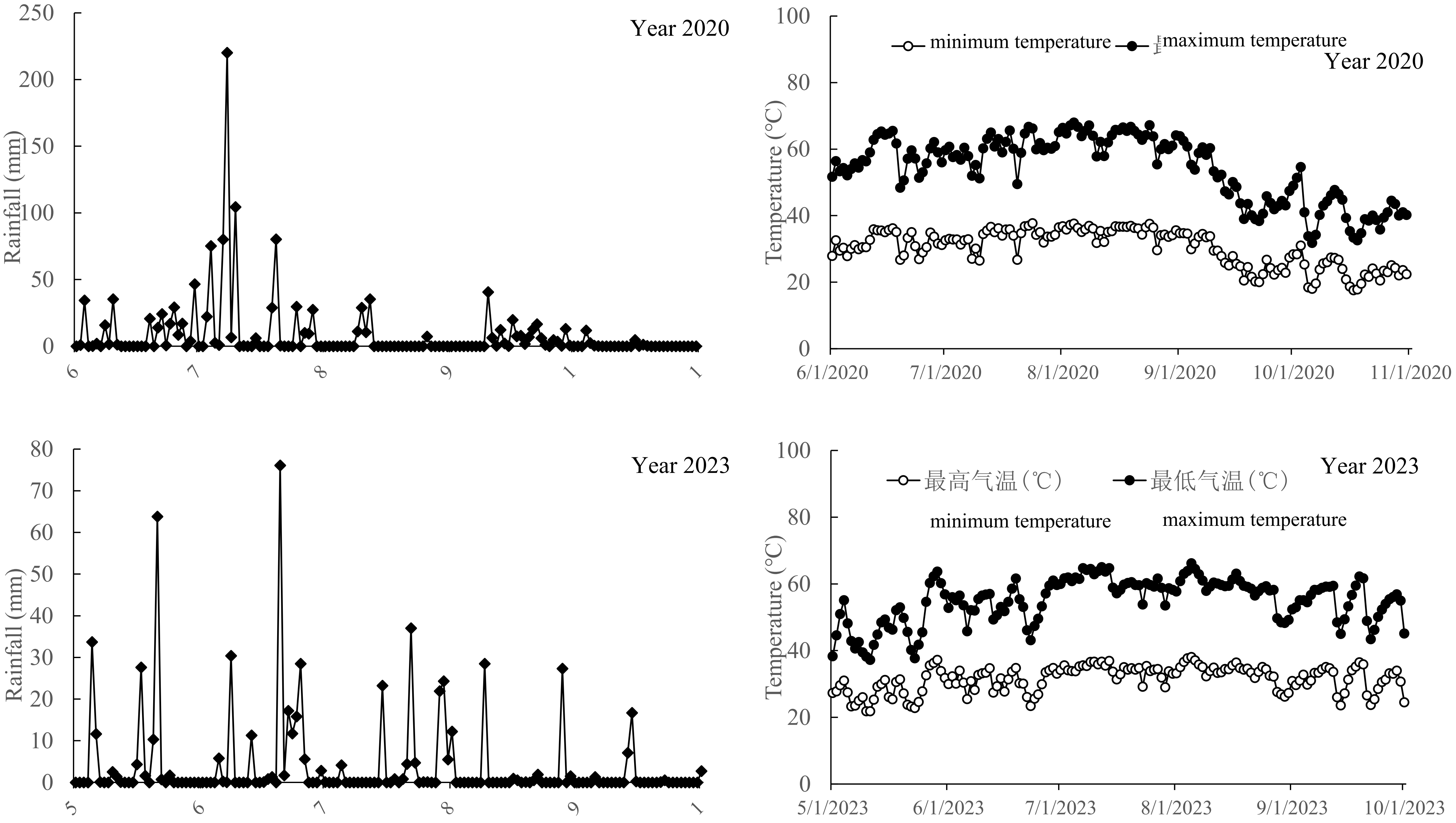

Figure S1. Daily maximum temperature (●), minimum temperatures(○) (2020: A; 2023: C)and rainfall(♦) (2020: B; 2023: D) during growing period

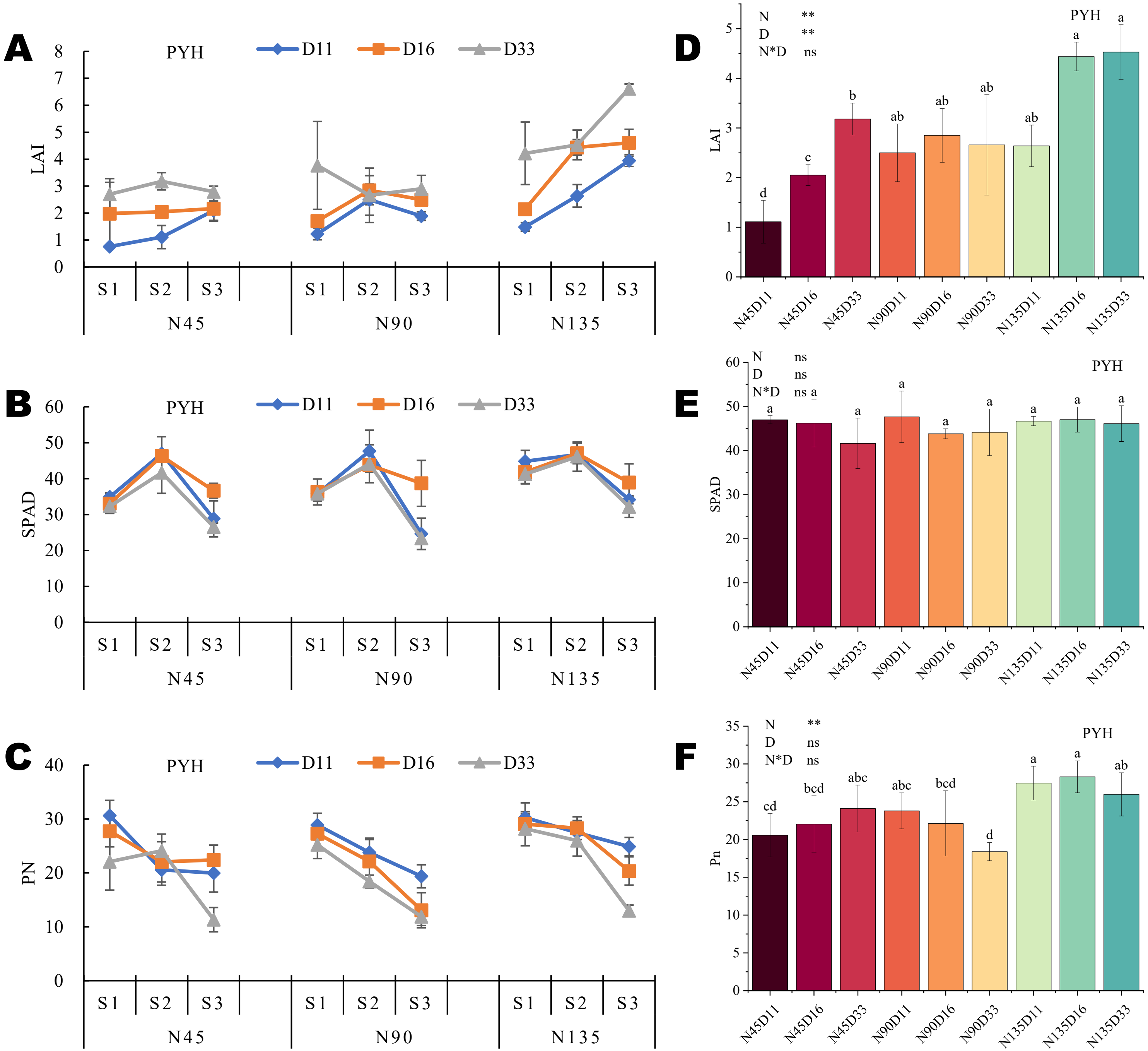

Figure S2. Interactive effects of nitrogen dose and planting density on leaf area index (LAI), chlorophyll content, and net photosynthesis (Pn) of black sesame variety PYH in 2023. (A) Dynamic LAI changes. (B) Dynamic chlorophyll content changes. (C) Dynamic Pn changes. (D)-(F) Variation of LAI, chlorophyll content, and Pn, respectively, under different planting densities and N doses. Note: N45, N90, and N135 represent the nitrogen application rate of 45, 90, and 135 kg · ha-1, respectively. D11, D16, and D33, and D33 represent planting density of 110000, 160000, and 330000 plants per ha-1, respectively. S1 represents the initial flowering period, three days after topdressing; S2 represents 20 days after topdressing; S3 represents 40 days after topdressing; and S4 represents 60 days after topdressing.

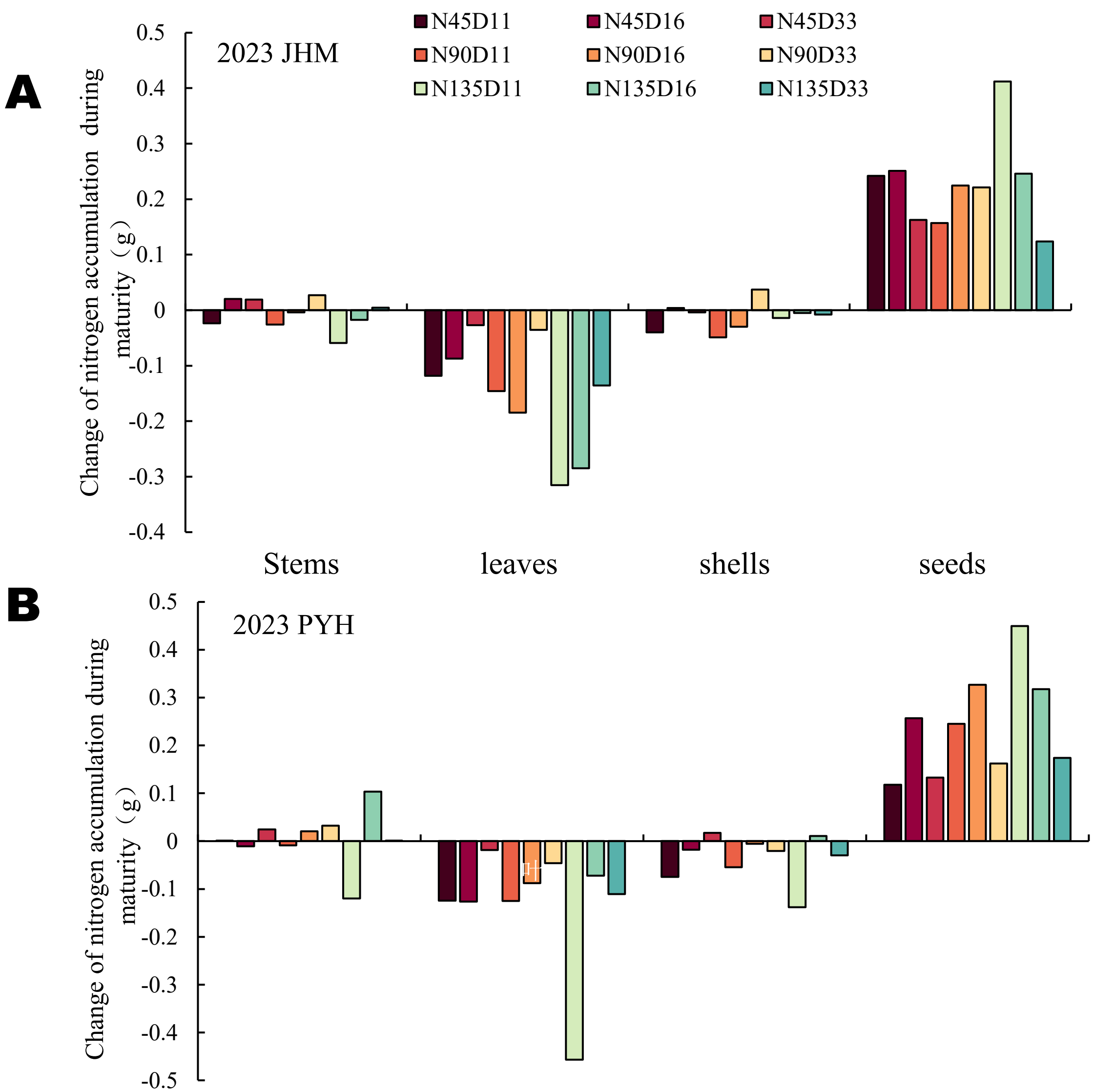

Figure S3. (A)-(B) Changes in nitrogen accumulation in various tissues from the late-flowering to the harvesting stage in JHM and PYH, respectively, in 2023.

Note: N45, N90, and N135 represent the nitrogen application rate of 45, 90, and 135 kg · ha-1, respectively. D11, D16, and D33, and D33 represent planting density of 110000, 160000, and 330000 plants per ha-1, respectively. S1 represents the initial flowering period, three days after topdressing; S2 represents 20 days after topdressing; S3 represents 40 days after topdressing; and S4 represents 60 days after topdressing.

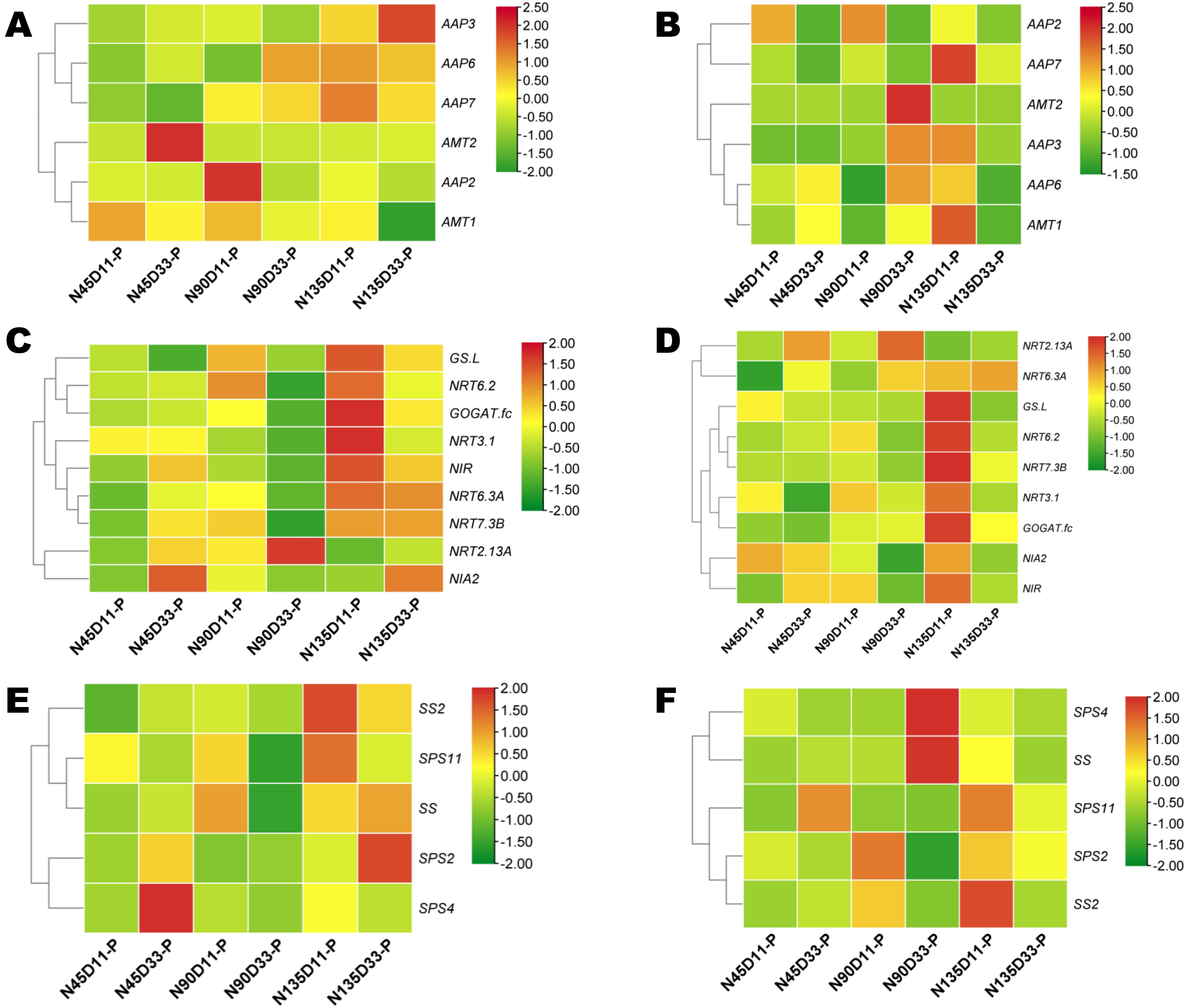

Figure S4. Interactive effects of nitrogen dose and planting density on the relative expression of N metabolism and sucrose metabolism-related genes in leaves of variety PYH in 2023. (A)-(B) Ammonium transporters at S2 and S3, respectively. (C)-(D) Nitrate transporters at S2 and S3, respectively. (E)-(F) Sucrose metabolism-related genes at S2 and S3, respectively. Note: N45, N90, and N135 represent the nitrogen application rate of 45, 90, and 135 kg · ha-1, respectively. D11, D16, and D33, and D33 represent planting density of 110000, 160000, and 330000 plants per ha-1, respectively. S1 represents the initial flowering period, three days after topdressing; S2 represents 20 days after topdressing; S3 represents 40 days after topdressing; and S4 represents 60 days after topdressing.

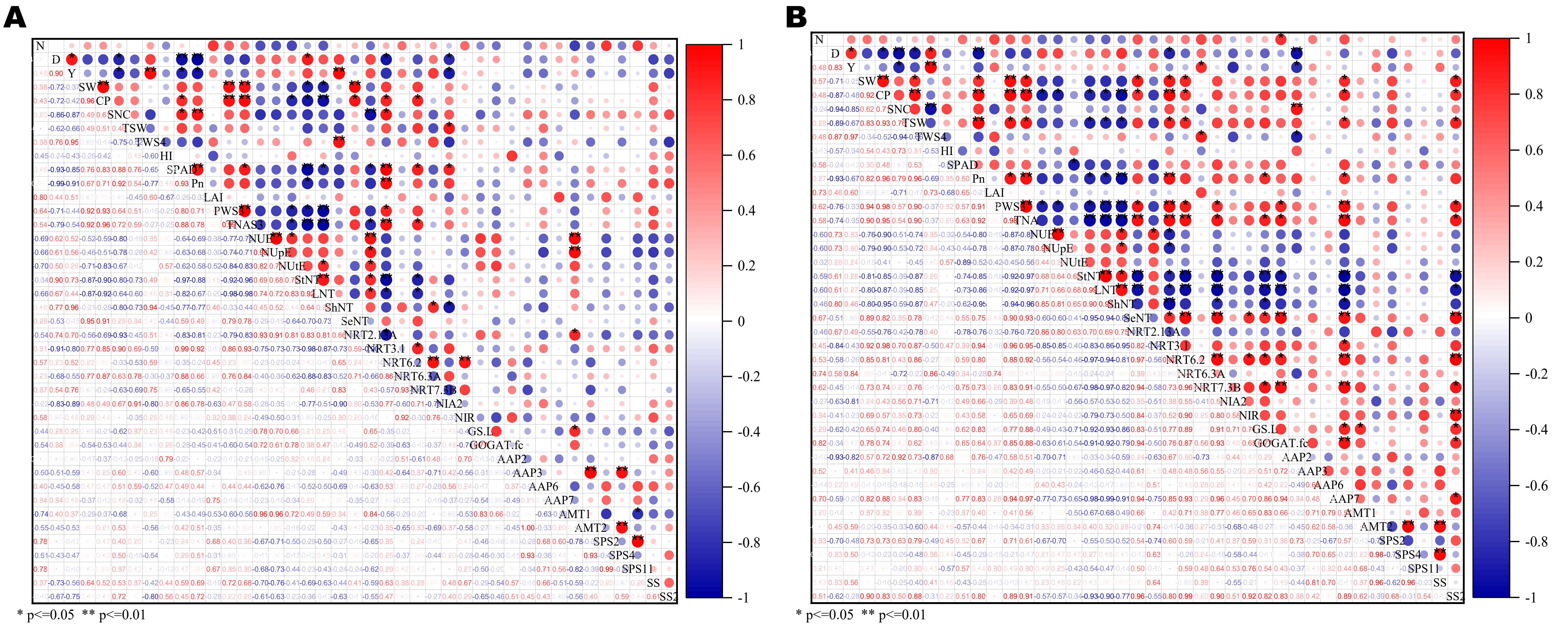

Figure S5. Correlation analysis of yield, nitrogen utilization efficiency, nitrogen transport, nitrogen and carbon metabolism-related genes' expression in leaves at the flowering stage in JHM (A) and PYH (B)
